# Supplementary figures and images for: Ubiquitin-protein ligase Ubr5 cooperates with hedgehog signalling to promote skeletal tissue homeostasis
Source: PLoS Genet. 2021 Apr 5;17(4):e1009275. doi: 10.1371/journal.pgen.1009275 (PMC8057592; doi:10.1371/journal.pgen.1009275)

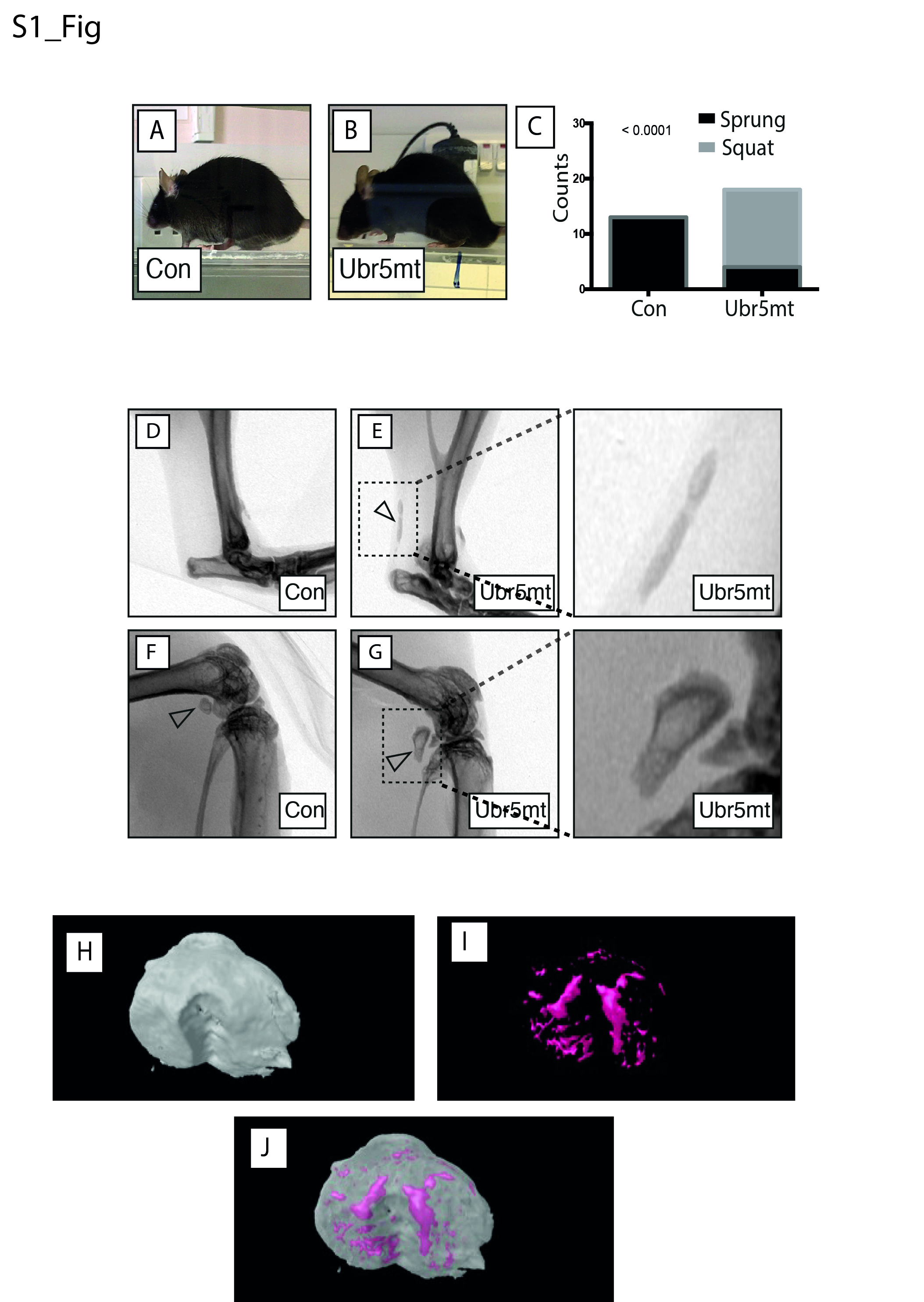

Supplement: S1 Fig — 24-week-old control or Ubr5mt mice were assessed for (A-C) behavioral analysis. (A,B) Mice were videoed while walking along a boxed runway and their static positioning recorded as either ‘sprung’ (with their posterior not in contact with the floor), or ‘squat’ (with their posterior resting on the floor). (C) Graph showing counts of animal behavior. n = six of each gender for controls and eight of each gender for the Ubr5mt genotype. Fisher’s exact test, p value = <0.0001. (D) Control ankles and (E) Ubr5mt ankles which exhibited ventrally- and dorsally located isolated signals. Dashed box region enlarged in indicated panel. (F) Control knee joints exhibited the fabella, a dorsally-located sesamoid bone (open arrowhead). (G) Ubr5mt knee joints exhibited a misshapen fabella (open arrowhead), with the dashed boxed region being enlarged in the indicated panel to the right). n = eight males and eight females. (H-I) show the volume rendered 3D models of 26-week-old tibial subchondral bone. Total subchondral bone volume used in the analysis is shown in grey (H) and the high-density signal in red (I) and the two merged (K). (TIF) [file pgen.1009275.s001.tif]

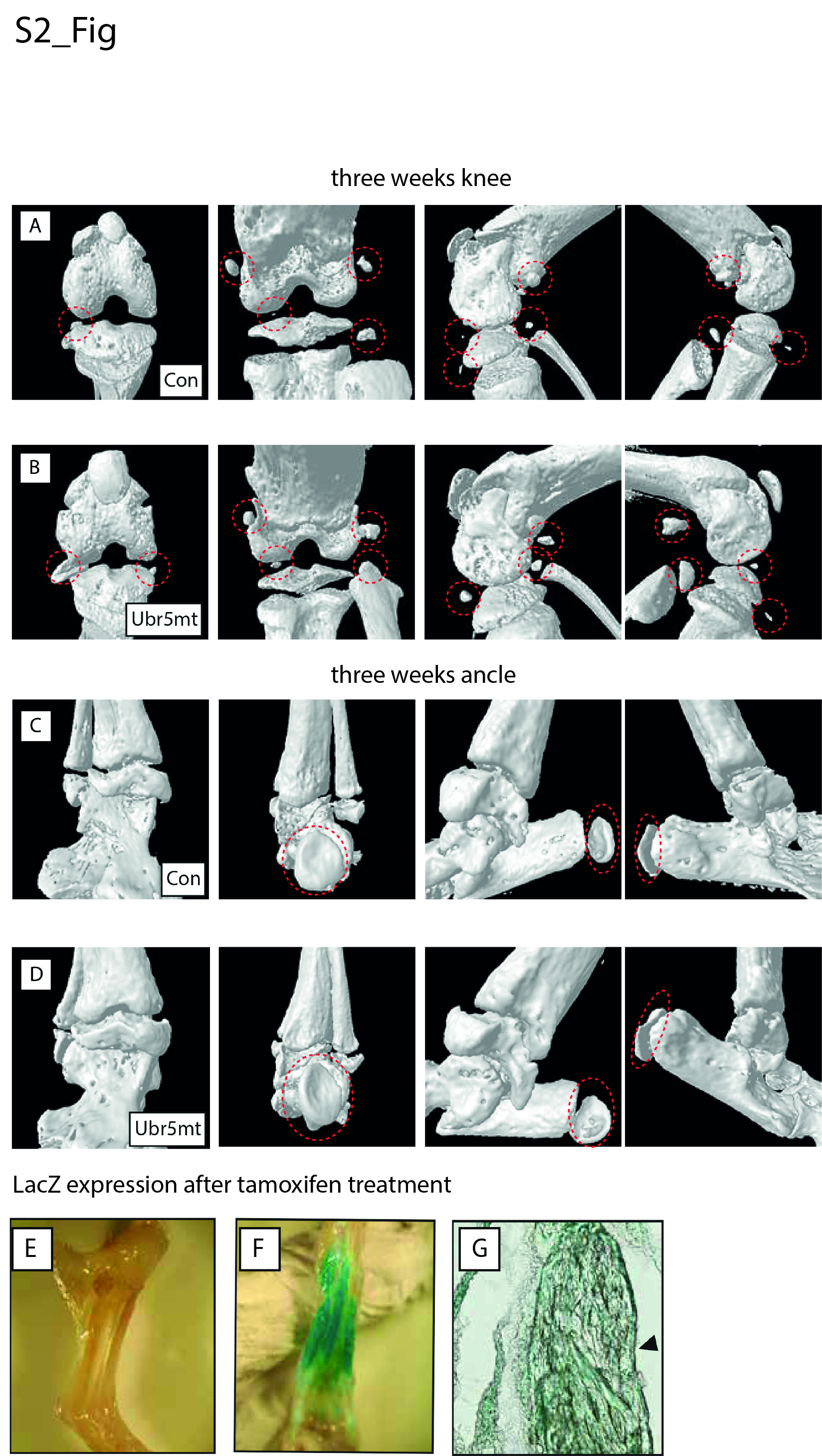

Supplement: S2 Fig — (A-D) Different views of surface rendered 3D models of three-week-old control and Ubr5mt (A,B, respectively) knee and (C,D, respectively) ankle joints. (A-D) From left to right panels: ventral, dorsal, medial and lateral views. Both control and Ubr5mt joints exhibit either a normal array of sesamoid bones, developing epiphysis and calcifying menisci. (E-G) Analysis of 18-week-old tamoxifen-treated pCAGG-Cre control and pCAGG-Ubr5mt ankle joints. (E,F) Whole mount β-Gal staining of (E) control and (F) pCAGG-Ubr5mt ankle joints reveals β-gal expression in muscles and associate tendons. Sagittal section of ankle joint (G) showing an ectopic structure associated with the AT midbody (closed arrowhead) stained positive for Ubr5/UBR5 expression. (TIF) [file pgen.1009275.s002.tif]

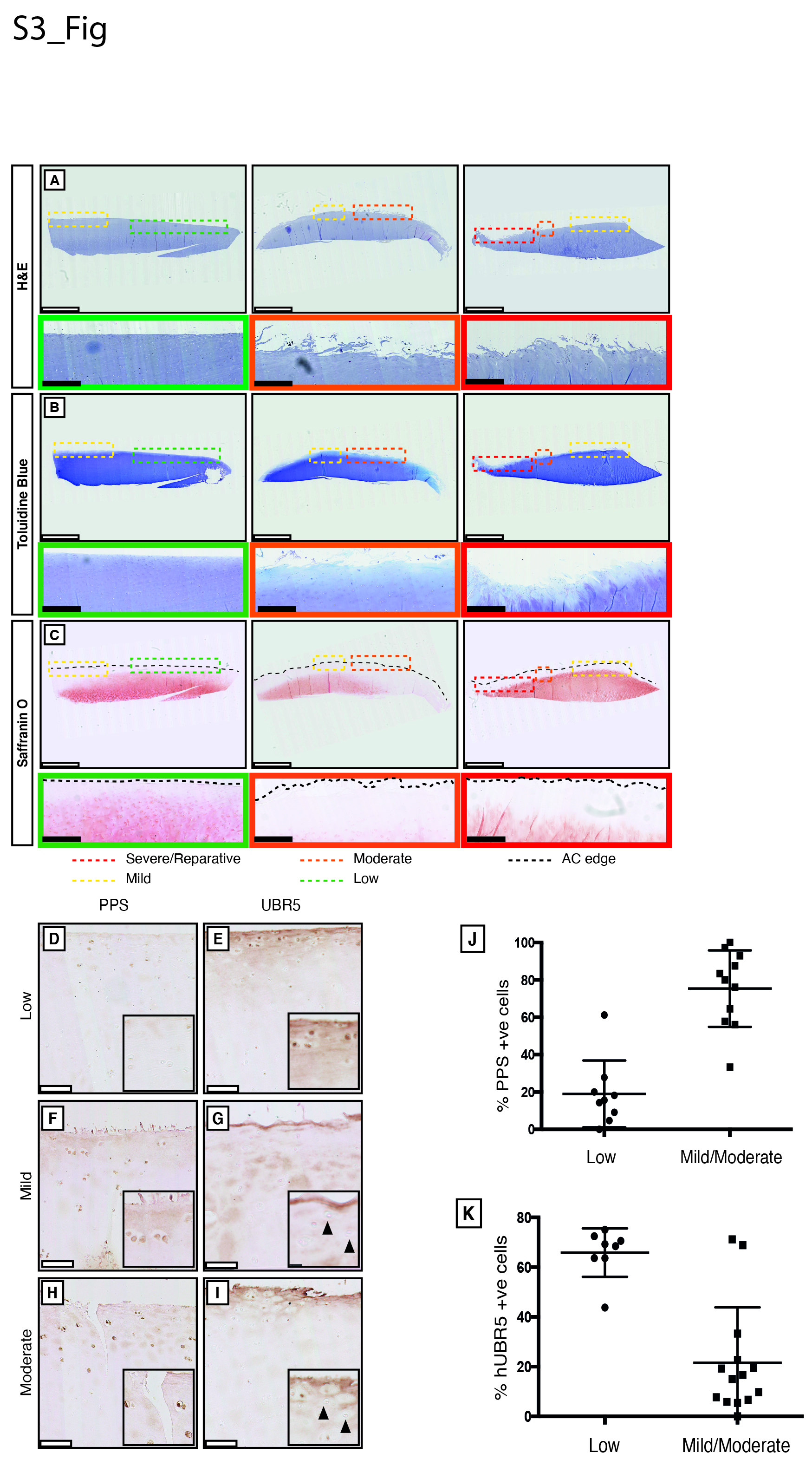

Supplement: S3 Fig — (A-C) Examples of human OA patient material stained with (A) haematoxylin and eosin (H&E), (B) toluidine blue or (C) safranin O revealed intra- and inter-sample variation in AC defects. Coloured boxes indicate regions of the varying OA severity (please see figure key). (A-C) Colour-coded, magnified dashed boxes in upper panels are shown in more detail in the colour-coded lower panels (thick outlines). Moderate-scored regions (orange) exhibited extensive surface fibrillation and reduced toluidine blue and safranin O staining in comparison to low-scored regions (green). Severe-scored regions (red) exhibited loss of safranin O staining and apical-basal clefts in the AC surface. (C) The dashed black lines indicate the apical edge of the AC. (D-K) Human AC samples graded as low, mild or moderately damaged were analysed for (D,F,H) PKA activity (PPS) and (E,G,I) UBR5 expression. Graphs of percentage of (J) PPS and (K) UBR5 positive cells for low and combined values for mild and moderate AC grades. Mean and s.e.m indicated. n = six biological replicates. Fishers exact test on pooled cell count data. p = <0.0001 for both. (TIF) [file pgen.1009275.s003.tif]

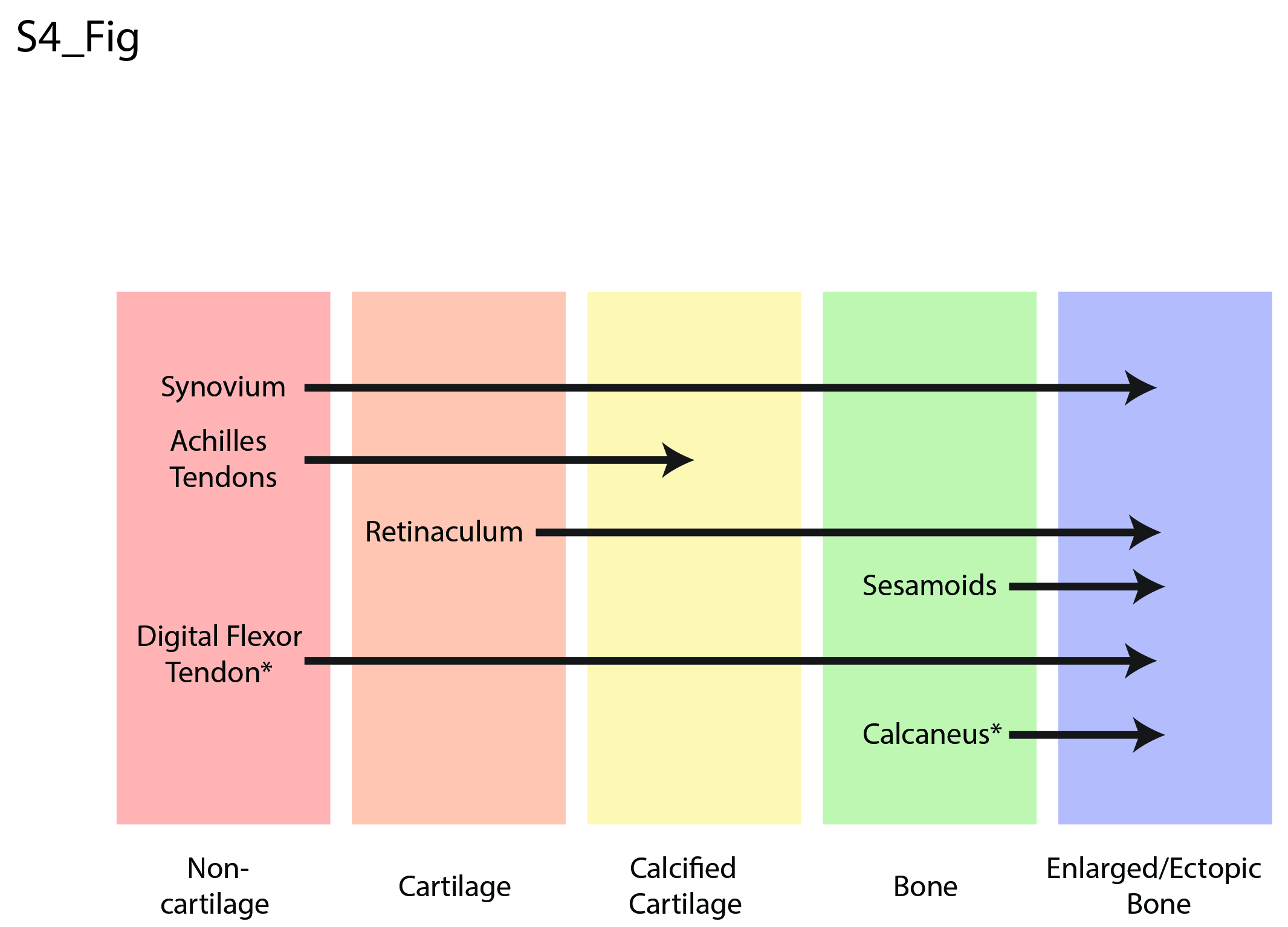

Supplement: S4 Fig — Overview of the metaplastic events of various Ubr5mt tissues. Red = non-cartilaginous tissues (Synovium, AT and Superficial Digital Flexor tendon); Orange = cartilaginous tissues (retinaculum); Yellow = calcified cartilage; Green = normotopic bone; Blue = heterotopic or enlarged normotopic bone. Arrows indicate the direction of metaplasia, with the arrowhead indicating the tissue type in 24-week-old Ubr5mt and/or Ubr5mt+SmoLoF animals. Metaplastic tissue events unique to Ubr5mt+SmoLoF are indicated by an asterisk. (TIF) [file pgen.1009275.s004.tif]
