# Supplementary material for: Ubiquitin-protein ligase Ubr5 cooperates with hedgehog signalling to promote skeletal tissue homeostasis
Source: PLoS Genet. 2021 Apr 5;17(4):e1009275. doi: 10.1371/journal.pgen.1009275 (PMC8057592; doi:10.1371/journal.pgen.1009275)
Supplement: Attachment — Submitted filename: The paper entitled.docx [file pgen.1009275.s005.docx]

The paper entitled “ Ubiquitin-protein ligase Ubr5 cooperates with Hedgehog signalling to promote skeletal tissue homeostasis” investigates the role of UBR5 in regulation of

the musculoskeletal system and its modulation of mammalian hedgehog signalling.

The paper represents a significant body of work encompassing several transgenic mice and *in vitro* cell work as well as human disease comparison. In fact to me this work should be divided in back-to-back papers addressing each of the organs affected in order to give an appropriate levels of specialism.

As it is, the authors have shown that Loss of the *Ubr5* gene in early limb mesenchyme resulted in postnatal defects in and around joints within the fore and hind limbs. This post-natal aberrant changes using two independent transgenic models is undeniable evidence. These changes were documented in µCT “signals” as they called them exhibited multiple ectopic calcification around the knee and ankle joints affecting tendons and skeletal muscle. As Ubr5 manifest itself post natally and increases with adulthood, I wondered whether the Ubr5 is induced by loading of the musculoskeletal system in limbs? Indeed, Ubr5 was the most significantly upregulated transcript across all models of loading/exercise (*Scientific Reports,2019* 9(1) 4251). It would be important for the authors to address this part as muscle is fundamental. In a couple of the publications, the tendons were not dissected out from the body of muscle in the analysis and can not be excluded from the function of musculoskeletal system as a whole.

Moreover, Ubr5 has been recently shown to be involved in skeletal muscle atrophy, recovery from atrophy and injury, anabolism and hypertrophy (PMID: 31093990) where the authors demonstrated that Ubr5 was epigenetically altered via DNA methylation during recovery from atrophy. These links should be addressed in the discussion.

In this regard, I wonder whether the NIH3T3 cells (skin fibroblasts) were the right cells to transfect to address the *in vitro* investigation rather than primary tenocytes or even human tenocyte cell lines?? As tenocytes were shown to have trans-differentiated into chondrocytes with the Loss of the Ubr5 gene, I am reminded of a recent paper Exp Cell Res 2012 318, Issue 13, 1492-1507, where sox9 converted tenocytes into chondrocyte. Does Ubr5 regulates either sox9 or scleraxis availability??

In addition to ectopic bone and cartilage formation, defects in articular cartilage was noted and degradation was substantial. Although the authors have shown timeline for the degradation, they assumed that this is osteoarthritis (OA), based on histology, without showing any immunohistochemistry of epitopes of at the surface of articular cartilage such as DIPEN or NVETGE for aggrecan or Col II degradative epitopes, nor did they show MMP13 associated with increased hypertrophic chondrocytes in articular cartilage. The question remains is this so called OA, a degradative as in classical OA or from bottom up similar to alkaptonuria where the calcified cartilage produces a fissure that goes upwards towards the articular surface.

The other point I would like to raise is the chondrocyte cilia with smo. The activation of smo involves ciliary enrichment. A recent paper (Dev Biol. 2018 May 15; 437(2): 152–162) suggested that proper ciliary smo enrichment is necessary for full smo activation. When the authors examined the addition of smo (loss of function) to the loss of Ubr5, they may have affected the ciliary transport and therefore had more severe outcome!

I agree with the authors that the Ubr5mt mouse model could assist in uncovering mechanisms that lead to disorders including characterisation of early pathological events and elucidation of pro-homeostatic mechanisms capable of promoting global mechanism. However, I feel that the suggested diagram in the (figure 9E) is a bit limited and perhaps “naïve” since AC would respond different from tendon, synovium or muscle. Indeed human OA has not shown hypertrophy as a mechanism, unlike mouse AC for example which I am sure the authors agree that it suggests different responses to the loss of Ubr5 in different cell types and this is why I feel that this paper may represent several stories that is common to the loss of Ubr5 but with different mechanism.
