# Supplementary material for: Ubiquitin-protein ligase Ubr5 cooperates with hedgehog signalling to promote skeletal tissue homeostasis
Source: PLoS Genet. 2021 Apr 5;17(4):e1009275. doi: 10.1371/journal.pgen.1009275 (PMC8057592; doi:10.1371/journal.pgen.1009275)
Supplement: Attachment — Submitted filename: PLoS_GENETICS_Reviewers120321.docx [file pgen.1009275.s006.docx]

Dear Editors-in–Chief,

We thank the reviewers for their constructive comments on our manuscript. We have attempted to answer all the questions raised and our responses are listed in order below. We now feel that the manuscript is now presented in a more straightforward fashion and the data is organized so that it will be easier for the readers to follow.

**Reviewer's Responses to Questions**

Comments to the Authors:

**Reviewer 1**

The paper represents a significant body of work encompassing several transgenic mice and in vitro cell work as well as human disease comparison. In fact to me this work should be divided in back-to-back papers addressing each of the organs affected in order to give an appropriate levels of specialism.

**We thank the reviewer for his kind words and in fact, we had initially divided this work into two papers one concerning ECCO and the other the AC defects. We finally decided that it was difficult to separate the phenotypes and to keep the papers from being somewhat redundant and therefore, now prefer this route.**

As it is, the authors have shown that Loss of the Ubr5 gene in early limb mesenchyme resulted in postnatal defects in and around joints within the fore and hind limbs. This post-natal aberrant changes using two independent transgenic models is undeniable evidence. These changes were documented in µCT “signals” as they called them exhibited multiple ectopic calcification around the knee and ankle joints affecting tendons and skeletal muscle. As Ubr5 manifest itself post natally and increases with adulthood, I wondered whether the Ubr5 is induced by loading of the musculoskeletal system in limbs? Indeed, Ubr5 was the most significantly upregulated transcript across all models of loading/exercise (Scientific Reports,2019 9(1) 4251). It would be important for the authors to address this part as muscle is fundamental. In a couple of the publications, the tendons were not dissected out from the body of muscle in the analysis and can not be excluded from the function of musculoskeletal system as a whole.

**The reviewer raises a good point, however it is not clear whether loading has an effect on Ubr5 expression in the cartilaginous tissue in the joints and we are not aware of publications that address this issue. Our data, of course, shows an increase in UBR5 expressing cells in human AC in disease (Fig. S3 Fig K). In mouse Prx1Cre would not lead to UBR5 deficiencies in muscle; however, the pCAGG-Cre is more broadly expressed. In these experiments, we only examined the connective tissue; but did not see overt defects in the muscle. So as the reviewer suggests determining the role of Ubr5 more broadly in musculoskeletal system but using different tissue specific Cre drivers would be of interest particularly in light of the recent papers (Seaborne RA, et al. J Physiol. 2019;597(14):3727-49 and Seaborne RA, et al. Sci Rep. 2018;8(1):1898. ).**

Moreover, Ubr5 has been recently shown to be involved in skeletal muscle atrophy, recovery from atrophy and injury, anabolism and hypertrophy (PMID: 31093990) where the authors demonstrated that Ubr5 was epigenetically altered via DNA methylation during recovery from atrophy. These links should be addressed in the discussion.

**We have added in the discussion a few sentences that bring the observations in muscle together with our data on Ubr5 in the joints. At line 321 we now state—**

**“Recently, increased *Ubr5* expression was correlated with muscle growth, hypertrophy [40] and recovery from injury [41] suggesting a role in muscle maintenance. These reports suggest that *Ubr5* may have a broad role in the musculoskeletal system in the limbs affecting homeostasis not only in the cartilaginous connective tissue but also in the associated musculature.”**

**The references used here are (Seaborne RA, et al. J Physiol. 2019;597(14):3727-49 and Seaborne RA, et al. Sci Rep. 2018;8(1):1898. ).**

In this regard, I wonder whether the NIH3T3 cells (skin fibroblasts) were the right cells to transfect to address the in vitro investigation rather than primary tenocytes or even human tenocyte cell lines?? As tenocytes were shown to have trans-differentiated into chondrocytes with the Loss of the Ubr5 gene, I am reminded of a recent paper Exp Cell Res 2012 318, Issue 13, 1492-1507, where sox9 converted tenocytes into chondrocyte. Does Ubr5 regulates either sox9 or scleraxis availability??

**We agree with the reviewer that it is often difficult to determine the correct experimental system to use. Here, we use NIH 3T3 cells for these studies because they are the best characterised in vitro system of canonical and non-canonical Hh signalling. Our collaborator and co-author (NRDG) is a leader in this field and has identified many Hh regulatory proteins using NIH 3T3 cells, findings that were later reported in other cell types and tissues (Riobo et al., Proc Natl Acad Sci U S A. 2006 Mar 21;103(12):4505-10; Riobo et al, 2006 Aug 15;103(33):12607-12; Polizio et al., J Biol Chem. 2011 Jun 3;286(22):19589-96). In fact, in this study we were able to reproduce the findings observed in Ubr5mut mice in shUbr5 NIH 3T3 cells, including the upregulation of cAMP/PKA signalling axis. Therefore, the need to generate immortalised Ubr5-knockdown tenocytes and to establish a robust Hedgehog signalling assay in those cells was considered unnecessary. The latter is not trivial, since in addition to needing to be fully competent for Hh signalling, the in vitro model needs to be amenable to stable transfection for selection of shRNA-stable clones, and subsequently to transient transfection with four plasmid combinations required for Gli-luciferase assays (empty or Hh signalling testing plasmid (such as Smo or Gli1), Gli-luciferase plasmid, Renilla-luciferase plasmid and plasmid carrying Ubr5 or empty control), and survive prolonged serum starvation for primary cilium formation. And lastly, the analysis of SOX9 and scleraxis in the mutant is an interesting question and is certainly an avenue worth pursuing.**

In addition to ectopic bone and cartilage formation, defects in articular cartilage was noted and degradation was substantial. Although the authors have shown timeline for the degradation, they assumed that this is osteoarthritis (OA), based on histology, without showing any immunohistochemistry of epitopes of at the surface of articular cartilage such as DIPEN or NVETGE for aggrecan or Col II degradative epitopes, nor did they show MMP13 associated with increased hypertrophic chondrocytes in articular cartilage. The question remains is this so called OA, a degradative as in classical OA or from bottom up similar to alkaptonuria where the calcified cartilage produces a fissure that goes upwards towards the articular surface.

**We thank the reviewer for pointing out the difficulties in showing that AC degradation in mouse corresponds to osteoarthritis without further investigations. We are aware of these difficulties and have attempted to take particular care in referring to the mouse AC phenotype as OA; however, we did want to draw parallels where they occurred. I have reviewed where we mention OA and have now changed the sentence at line 88 that read	“These findings may influence current therapeutic approaches modulating HH signalling for the treatment of osteoarthritis and heterotopic ossification.” To now read	“These findings may influence current therapeutic approaches modulating HH signalling for the treatment of degenerative musculoskeletal conditions such as osteoarthritis and heterotopic ossification.”**

The other point I would like to raise is the chondrocyte cilia with smo. The activation of smo involves ciliary enrichment. A recent paper (Dev Biol. 2018 May 15; 437(2): 152–162) suggested that proper ciliary smo enrichment is necessary for full smo activation. When the authors examined the addition of smo (loss of function) to the loss of Ubr5, they may have affected the ciliary transport and therefore had more severe outcome!

**The reviewer raises an interesting point about the molecular basis of the ‘sensitized’ Hh pathway background. The *Smo* loss of function mutation used here is an exon1 replacement by homologous recombination (ref 31 also, Zhang et al. *Cell* 105, 781-792 [2001]) so our assumption, here and the most likely scenario is that there is a reduction in the levels of SMO in the cell. It is not clear how this would impact the cilia and whether in combination with UBR5 has a discernible ciliary phenotype but, as the reviewer suggests, is an interesting research avenue to go down.**

I agree with the authors that the Ubr5mt mouse model could assist in uncovering mechanisms that lead to disorders including characterisation of early pathological events and elucidation of pro-homeostatic mechanisms capable of promoting global mechanism. However, I feel that the suggested diagram in the (figure 9E) is a bit limited and perhaps “naïve” since AC would respond different from tendon, synovium or muscle.

Indeed human OA has not shown hypertrophy as a mechanism, unlike mouse AC for example which I am sure the authors agree that it suggests different responses to the loss of Ubr5 in different cell types and this is why I feel that this paper may represent several stories that is common to the loss of Ubr5 but with different mechanism.

**We have attempted in our discussions of the Ubr5 mutant phenotype to keep the analysis of the ECCO clearly defined and separate from that of the degradation of the AC; however, these do share a common signaling mechanism. We do agree that these data could have been split into two separate papers but we feel this route will give the reader a general overall assessment of the Ubr5 degenerative phenotype.**

**Reviewer #2:**

General comments

In summary I would say this manuscript adds knowledge to this area, its, perhaps apparently paradoxical and at times slightly superficial and descriptive findings, are worthy of publication. It is extremely important that all information, including that which is mechanistically unresolved or at apparent odds with dogmas, is in the research domain.

Fundamentally there is multi-faceted analysis, sometimes unnecessarily quantitated, to support the major descriptive findings in the mouse backed up by follow-up in vitro studies. Importantly, I am in agreement with the central tenets of the manuscript as summarised, they are supported by the evidence, but I feel that for maximal impact the manuscripts readability could be markedly improved.

**We thank the reviewer for these comments and have taken this opportunity to make changes, particularly in the first three sections of the results that should make reading the manuscript more straightforward without changing the context of these sections**

This is often points of clarity, improvements to figures and legends etc, but also the authors may wish to consider the salient findings and decluttering the main figures- as in current form the paper is very dense and not easy to follow. The authors are to be commended for a valiant attempt to mechanistically explain the mechanistic links to Hh and PKA signalling in vitro which add support to interpretations of the findings.

**We have taken the reviewers suggestion and have reorganized figures 1-5 which should make it easier for the reader to navigate.**

As the authors note it is of particular interest that the phenotypes are only apparent in maturing or mature context despite the developmental nature of the pertubation. What do the authors propose might be the reason for this? Does an evolving landscape of Hh signalling account for this and/or are their mechanical inputs into this regulatory axis? Perhaps linkable to locations of ectopic ossification/ failure of cartilage at interface with bone (tears).

**The reviewer raises a number of issues that are important to pursue. Our experiments showed that Ubr5 is required postnatally (time course of live animals and the pCAGG-Cre ERT2) and highlighted that the overt expression of the phenotype is not detectable until 6 weeks and from that stage on is progressive. Perhaps a minor, subtle role for Ubr5 may exist during development but as we previously published, there is no discernible input into Hh signaling at these early stages. We present data that suggest a role for UBR5 in maintaining tissue homeostasis interfacing with Hh signaling at postnatal stages. In the absence of UBR5 impairment of homeostatic function leads to aberrant tissue repair, and the accumulation of tissue damage Biomechanical stress is a likely source that challenges these homeostatic functions leading to a cycle of damage and aberrant repair. The histopathological defects in older tissues appear as heterotopic ossification, and metaplasia and shedding of AC as a consequence of aberrant Hh signaling. These are important research avenues to pursue and the approaches would include further genetic analysis such as the use of more conditional mutants with highly tissue specific drivers and biochemical analysis to unravel the components of the affected pathways. Thus, a further, substantial amount of work is required which we feel is outside the scope of this study. However, these join a long list of questions that need to be explored further.**

I wasn’t clear throughout – are the effects limited to hind limbs? Do you have images of forelimbs?

**The hindlimb phenotype was much more severe than that in the forelimb, perhaps as a consequence of differences in load bearing; therefore, all of our work concentrated on the more severely affected hindlimbs.**

Was cartilage thickness measured- were effects bias to compartments ?

**Histological sectioning across the lateral-medial axis of young animals (3 weeks) revealed no dramatic differences in cartilage thickness. Unfortunately, an inability to visualise and volumetrically quantity such tissues by uCT prevented an accurate assessment.**

**In older UBR5mt cartilage, the cracks within and shedding of cartilage appeared exclusively within the middle of the section along the lateral-medial axis. The sagittal section in Figure 6M clearly indicates the focal loss of cartilage within the “middle” of the tibial cartilage and its preservation at the edges.**

Is cartilage loss a consequence of changes to other soft tissues in limb?

**This is an important question. Due to the physical interconnectivity of the different tissue within and surrounding joints we cannot establish tissue-specific cause and effect. For example, defects in tendon function may destabilise their associated joints, which in turn causes increases biomechanical loading of cartilage and its degradation.**

**To address such a hypothesis is an aim of follow-up work and would require the development of exquisitely tissue-specific Cre-ER-T2 driver lines.**

Changes to gait – do mice move apparently normally (beyond squat resting position).

**We did not systematically investigate these questions. The squat position was the most easily observed and identifiable phenotype especially with increasing age. In addition, the only differences in the gait of these animals that we observed was that the mutants had a tendency to carry their hindpaw digits in a more splayed position.**

Were mice weights normal ? Perhaps previously described.

**Over the period of these studies the mice were routinely weighed and the mutant mice were not significantly different from the controls. This is now stated in the Materials and Methods section lines 397-398.**

What were genders of small groups (n=3)

**Unless otherwise stated, males were used. This is now stated in the Materials and Methods section lines 397-398.**

Minors:

I found Figure 1 early panels extremely hard to navigate. Line 108 are ankles shown?

**Figure 1 has been reorganized and should be easier for the reader to follow. In doing so we removed panel G, which was a higher magnification of F and didn’t add significant information to the figure**

**Line 108. We thank the reviewer for pointing this out; we are only showing data in Fig1 a, b of the knee so the line 108 has been changed to reflect this.**

Line 120 authors conveying this shows us where UBR5 is ? - consider re-phrase plain English

**Line 120 now sentence including lines 119-123 has been added to be more readable**

Lines 123 beta-gal activity, thus they are derived from embryonic mesenchyme ?

**Line123 (now incorporated into lines 119-125) changed to clarify the derivation of the β−gal expressing tissue.**

Line 134 different to what?

**Line134 has been changed so that it is now less ambiguous**

Lines 135-137 - no comparative controls to aid reader to see 'ectopic' - danger the non-specialist reader is completely lost

**Starting now on line 133. We have reorganized Figure 2 (now, A-F) which more directly focuses the reader’s attention to the ectopic structures and on line 132 refers back to Fig 1 to orientate the reader with the the ectopias.**

Line 145 “joints to reveal”

**Line 145. This sentence (now starting at line 137) has been changed**

Concern over n throughout in vivo studies (Limitations should be noted in discussion). No control for cage to cage variability possible with such low n. N could be more easily found for some studies too (key result descriptor- see ARRIVE guidelines). N is often 3 animals ?

**As suggested by the reviewer we have noted the limitations of our studies in describing the extent of the phenotypic variability and have added these sentences to the discussion. At line 293 in the first paragraph of the discussion we now state—**

**“The mutant phenotype displayed a degree of variability between individual mice; however, the abnormalities were pervasive in the mutants and were never observed in wildtype or control mice. The defects that were consistently observed included ectopic bone and cartilage formation, and articular cartilage degradation. The spectrum of defects observed are summarized in S4 Fig.”**

Legends are, however, detailed throughout suggesting good accordance with ARRIVE.

At times completely unclear as to n (e.g supplementary Figure 1 legend concerning gender breakdown for C).

**Supplementary figure 1 legend now reads “(C) Graph showing counts of animal behavior. n = six of each gender for controls and eight of each gender for the *Ubr5^mt^* genotype.”**

Figures – often at times magnifications are not matched (Fig 3H/I) , controls are missing, more labelling would help reader. Quantitation does not immediately tally with images (e.g high density regions in Fig 3B-F, and quantitation in G – 10-15% red?)

**As stated above, we have taken the reviewers suggestion and made a number of changes to the figures. In figure 3 we have added a control for comparison to the mutant which are now Fig 3 H, I. We have described in more detail the quantification of high density subchondral bone and added more information in the Material and methods section (Lines 433 - 449). We have also added a supplementary figure (S1 Fig J - L) that depicts the density analysis relative to the total volume of the subchondral bone.**

K is an example of unnecessary quantitation as also seen in Fig 5G ?

**Fig 3 K was removed**

Fig 4 N and O – please label up – what are we looking for? How was the data for 4Q generated?

**Figure 4N and O have been labelled**

Commend authors on correct use of Fisher's tests for contingency data.

**Reviewer #3:**

Specific comments:

1) I think the phenotyping done in here is excellent and I’m happy with this analysis of the paper

2) As the mechanistic analysis is surprising eg the fact that smoothened loss exarcerbated but did not suppress the phenotype, the key i think is how the loss of function of smoothened affected the gli induction.. It is clear that GLI is up in the UBR5 deleting animals so does Smoothened loss inhibit this. This need to be quantified (eg similar to work in figure 7)

**This analysis became particularly problematic due to the difficulty in obtaining sufficient high quality RNA material from the compound mutant cartilage. We attempted to quantify differences in Gli and several other markers using qRT-PCR. These analyses are difficult and require skilled dissection of soft tissue. While we were able to obtain sufficient number of samples of high quality RNA to show significant differences between UBR5 and controls (Fig 7 M), the SmoLoF X Ubr5^mt^ mouse cross generated fewer animals and the differences in Gli and (in Ptch1) between the compound mutant and the Ubr5mt showed no statistically significant differences** **These results, we feel, highlight the uncoupling of the increased phenotypic severity from any perturbation in a readout of canonical Hh signalling.**

3) in terms of the mechanism, have the authors looked at forskolin in vivo?

**We did consider this, but were concerned over interpretability as most of the 750+ GPCR (https://pubmed.ncbi.nlm.nih.gov/12761335/) utilise adenylate cyclase. Therefore, we felt the application to perform these mouse experiments in vivo would be difficult to justify.**

4) Likewise are the changes in any of the proposed pathways in figure 8E altered in the tissue e.g BRD4 etc...? It would help if the author could integrate their findings 3T3 cells with their in vivo tissue analysis.

**These are excellent suggestions and are future directions for this project. Unraveling the molecular mechanisms of UBR5 action would include investigating Ubr5 targets, their input into cell signaling and as suggested, analysis of candidates that participate in the noncanonical pathway. However, these are major research endeavours and we feel are outside the scope of this paper but hope these will in the future be the subject of further communications**
